# New Developments in the Treatment of IgG4-Related Disease: A Comprehensive Clinical Approach

**DOI:** 10.3390/jcm14196774

**Published:** 2025-09-25

**Authors:** Andrés González-García, Grisell Starita-Fajardo, David Lucena López, María Pilar Iranzo Alcolea, María López-Paraja, Mercedes Peña-Rodríguez, Francisco Lirola Sánchez, María Sánchez, Adrián Viteri-Noël, Martin Fabregate-Fuente, Mónica López-Rodríguez, José Luis Calleja-López, Luis Manzano Espinosa

**Affiliations:** 1 Systemic Autoinmmune Diseases Unit, Internal Medicine Department, Hospital Universitario Ramón y Cajal, IRYCIS, 28034 Madrid, Spainmlparaja@salud.madrid.org (M.L.-P.); luis.manzano@uah.es (L.M.E.); 2 Faculty of Medicine and Health Sciences, Universidad de Alcalá (UAH), 28801 Madrid, Spain

**Keywords:** IgG4-related disease, fibrosis, immune dysregulation, inebilizumab, obexelimab, rilzabrutinib

## Abstract

Immunoglobulin G4–related disease (IgG4-RD) is an uncommon fibro-inflammatory process characterized by the infiltration of tissues and organs and a typically dramatic response to glucocorticoids. Its relapsing–remitting course, multisystemic involvement, and variability in epidemiological and prognostic features pose a significant diagnostic challenge for clinicians. Despite their effectiveness in symptom relief, prolonged glucocorticoid use remains a challenge in IgG4-RD management, prompting the search for steroid-sparing alternatives. Although rituximab has recently demonstrated efficacy in the treatment of IgG4-RD, no consensus exists regarding the optimal maintenance regimen. The emergence of new B-cell–targeted therapies and other immunomodulators represents a promising step toward more personalized treatment approaches. In this review, we provide an updated and integrative overview of the emerging treatment strategies for IgG4-RD, highlighting future directions towards individualized management.

## 1. Introduction

Immunoglobulin G4–related disease (IgG4-RD) is a rare, chronic systemic fibroinflammatory disease that can affect multiple organs and lead to organ damage [1]. This condition was first identified at the beginning of the 21st century [2]. However, clinical presentations known today as classic features of IgG4-RD had appeared in the medical literature much longer before then [3]. Historically under-recognized, IgG4-RD is now increasingly diagnosed, and recent population-based data have begun to clarify its epidemiology. A claims-based study in the US estimated the incidence at 1.39 per 100,000 person-years with a point prevalence of 5.3 per 100,000 persons [4]. Histopathologically, it is characterized by fibrosis, obliterative phlebitis and lymphoplasmacytic infiltration in affected tissue infiltrate rich in IgG4-positive plasma cells; it often presents a relapsing–remitting course [1]. Nevertheless, the clinical heterogeneity of IgG4-RD, including variable organ involvement, differences in relapse risk and long-term morbidity, constitutes a diagnostic and therapeutic challenge for clinicians.

Despite glucocorticoids being the primary treatment for inducing remission, prolonged use is related to serious adverse effects and a high risk of relapse upon tapering or withdrawal [5]. Conventional immunosuppressants are often employed as steroid-sparing agents, but evidence supporting their efficacy is limited. Rituximab, a B-cell–depleting anti-CD20 monoclonal antibody, has emerged as an effective treatment option in both refractory and relapsing IgG4-RD [6]. Nevertheless, optimal dosing schedules, maintenance strategies, and long-term safety remain unresolved. This underlines the need for further research into the potential efficacy of glucocorticoid-sparing agents [7].

Recent advances in the understanding of IgG4-RD pathogenesis, particularly the role of B cells, plasmablasts, T follicular helper cells and fibrotic pathways, along with the recent development of novel targeted therapies, have paved the way for new treatments for the disease [8,9]. This review aims to provide a comprehensive overview of current and emerging therapeutic approaches to IgG4-RD within the context of the evolving understanding of its immunopathogenesis. To date, few studies have integrated emerging immunopathogenesis with the clinical applicability of therapies in advanced stages of development. This manuscript provides both a panoramic and forward-looking perspective that transcends a mere literature review, positioning itself at the intersection of basic science and clinical practice. We discuss the limitations of existing therapies, review clinical data on novel agents under investigation, and outline potential future directions toward personalized treatment strategies. Clinical trials discussed in this review were identified through a literature search of PubMed/MEDLINE and the ClinicalTrials.gov registry, focusing on interventional studies relevant to IgG4-RD published up to July 2025.

## 2. Clinical Spectrum and Therapeutic Challenges

The wide range of manifestations of IgG4-RD can lead to difficulties in establishing a diagnosis. Therefore, a high level of suspicion regarding IgG4-RD is necessary in order to avoid diagnostic delays and pitfalls. The four most commonly affected sites constitute the main phenotypes: head and neck involvement (including the salivary and lacrimal glands), pancreaticobiliary disease (e.g., autoimmune pancreatitis and sclerosing cholangitis), retroperitoneal fibrosis and aortic involvement, and systemic or multiorgan disease with lymphadenopathy, lung dysfunction, kidney disease, and/or central nervous system manifestations [10]. Although some patients present with a single-organ involvement, multisystemic disease is common and may develop over time, complicating diagnosis and follow-up [11].

The clinical course of IgG4-RD is typically chronic and relapsing–remitting. While many patients experience dramatic responses to glucocorticoid therapy, a substantial proportion relapse during dose tapering or after discontinuation. Recurrence rates of up to 40–50% have been reported, particularly in those with multiorgan involvement, elevated serum IgG4 levels, persistent plasmablast elevation, or certain organ patterns such as periaortitis [12,13,14,15,16]. The persistence of the disease burden increases the risk of progressive tissue damage and irreversible organ deterioration [12].

The clinical manifestations of IgG4-RD frequently mimic malignancy, infection, or other autoimmune diseases [17]. Consequently, it can lead to unnecessary invasive procedures or delays in diagnosis [18]. Moreover, the absence of pathognomonic biomarkers further complicates timely recognition [19]. Diagnosis relies on clinical suspicion of tumefactive organ involvement, radiological findings suggestive of enlargement or mass lesions, compatible laboratory data, and histopathology showing IgG4-rich lymphoplasmacytic infiltrates, storiform fibrosis, and obliterative phlebitis, though not all criteria are necessarily present [20]. Therefore, an integrated clinical-pathological diagnosis is essential.

From a therapeutic point of view, the heterogeneity of organ involvement and the absence of uniform criteria for initiating or maintaining treatment represent major challenges. Corticosteroids remain the first-line therapy for induction of remission [5]. However, long-term exposure is associated with significant adverse effects, most notably in older adults, who comprise the majority of patients with IgG4-RD [21]. The risk–benefit balance of chronic immunosuppression is particularly delicate in patients with indolent disease versus those at risk for organ-threatening damage.

In an effort to reduce the use of corticosteroids, strategies involving conventional immunosuppressants were frequently employed to reduce the risk of exacerbations, but robust, controlled data supporting their efficacy is lacking. The limited effectiveness of these agents in preventing relapse has led to a growing interest in biologic therapies, particularly B-cell–depleting agents.

## 3. Immunopathogenesis as a Therapeutic Framework

The pathogenesis of IgG4-RD is complex and only partially understood. It involves a unique interplay between innate and adaptive immunity, culminating in chronic inflammation, tissue infiltration by IgG4-positive plasma cells, and progressive fibrosis [9,22,23]. This immunopathological framework provides the basis for most therapeutic strategies currently in use or under investigation.

B-cell dysregulation is increasingly recognized as a pivotal pathogenic feature. The lymphoplasmacytic infiltrate together with an elevation in serum immunoglobulin concentrations, including IgG4, is frequently observed [24,25]. A hallmark of IgG4-RD is the expansion of an oligoclonal circulating population of CD19^+^CD20^−^CD27^+^CD38^++^ plasmablasts, which correlate with disease activity and relapse risk [26,27]. This B-cell activation is likely mediated through antigenic stimulation, but also involves aberrant interactions with T follicular helper (Tfh) cells and the overproduction of certain cytokines [9].

An increase in the number of CD4+ cytotoxic T lymphocytes is a key feature of the disease. These T cells express mediators of toxicity, profibrotic cytokines and SLAMF7, which are expanded in IgG4-RD and decrease during treatment [28]. This fibrotic component differentiates IgG4-RD from purely inflammatory autoimmune conditions and poses additional therapeutic challenges. Although no therapies currently target this subset specifically, their central role in fibroinflammatory damage makes them an attractive candidate for future therapeutic development. Among the most relevant cytokines produced by the Tfh, interleukin (IL)-4 and IL-10 are involved in B-cell class switching to IgG4, while transforming growth factor-β (TGF-β) contributes to the fibrotic response [22]. IL-21, produced by Tfh cells, plays a pivotal role in germinal center formation and plasma cell differentiation [29]. Elevated levels of BAFF (B-cell activating factor) have also been observed, suggesting a broader landscape of B-cell survival signaling in IgG4-RD [30].

The innate immune system may also contribute to disease pathogenesis. Eosinophilia is observed in a subset of patients, and increased serum IgE levels suggest Th2 skewing in some disease phenotypes [31]. While the overexpression of IgG4 provides a diagnostic clue, its pathogenic relevance remains controversial. Some authors propose that IgG4 may be a marker of chronic antigen exposure rather than a directly pathogenic immunoglobulin. Conversely, it has been suggested that elevated IgG4 levels may be simply an epiphenomenon [32]. However, it has already been noted that these antibodies also appear in other disease models, such as myasthenia gravis and pemphigus, where they have been responsible for causing damage [1].

Importantly, the immune pathways involved in IgG4-RD are not uniform across all patients. There is growing interest in the concept of immunological “endotypes,” where predominant pathways may vary according to the organ involved, disease stage, or underlying immunogenetic background [10,33]. This variability has direct implications for therapy: while B-cell depletion may be effective in most cases, some patients may benefit more from anti-cytokine therapy (e.g., IL-4/IL-13 inhibition) or from broader immunomodulation using JAK-STAT inhibitors. Emerging data also suggest a role for Bruton’s tyrosine kinase (BTK), a critical component of B-cell receptor signaling. BTK inhibitors may have therapeutic relevance in IgG4-RD by suppressing B-cell activation, plasmablast expansion, and possibly fibrogenic signaling, although clinical evidence remains limited at this stage [34].

## 4. Current Treatment Landscape

The management of IgG4-RD has traditionally relied on empirical immunosuppressive approaches, with glucocorticoids forming the foundation of therapy. In exceptional cases, a “watchful waiting” strategy has been suggested, whereby no treatment is initiated [5]. When selecting the most appropriate therapeutic strategy, numerous aspects should be carefully assessed, including the extent of disease, the severity and functional relevance of organ involvement, symptom burden, serological activity (e.g., elevated IgG4, hypocomplementemia or plasmablasts levels), and the risk of relapse [5]. Comorbidities, patient age, and prior exposure to immunosuppressive therapy should also inform therapeutic decision-making [35]. For example, certain manifestations such as retroperitoneal fibrosis, renal involvement, or biliary obstruction may require prompt and aggressive treatment to prevent irreversible organ damage, whereas other presentations may allow for a more conservative approach [1,5,19].

As a result of an international collaborative effort, the International Consensus Guidance Statement on the management and treatment of IgG4-RD was released in 2015 [5]. It remains the only widely accepted framework for therapeutic decisions in this condition based on expert opinion. The document outlined general treatment indications, recommended induction and maintenance regimens, and emphasized the need for long-term follow-up. However, its guidance predated the emergence of targeted therapies. European experts developed a therapeutic algorithm following the Delphi methodology, which also provides guidance on the structured management of the disease [35]. Furthermore, several studies have since expanded the range of treatments available for patients with IgG4-RD, introducing new options not covered in the original recommendations [36,37,38,39].

### 4.1. Glucocorticoids

Glucocorticoids remain the cornerstone of initial treatment for IgG4-RD and are usually effective at rapidly inducing clinical and radiological remission [1]. Most patients show a dramatic response within days to weeks of starting therapy, with marked improvement in symptoms, reduction in lesion size, and normalization of serum IgG4 levels [40,41]. This characteristic sensitivity to glucocorticoids has long been considered a hallmark of the disease, supporting the clinical diagnosis in typical cases [5]. A lack of response to a course of glucocorticoids suggests an alternative diagnosis should be sought, and this is considered an exclusive criterion in the ACR/EULAR 2019 classification [17]. Nonetheless, it should be acknowledged that, in certain circumstances, organ damage may already have occurred by the time of diagnosis and treatment is unlikely to reverse all baseline damage.

Induction regimens vary across clinical settings and geographical regions, but a commonly used approach consists of oral prednisone at an initial dose of 0.6 mg/kg/day for 2–4 weeks, followed by a gradual taper over the following months [5]. In some cases, particularly those with organ-threatening disease or multi-organ involvement, intravenous pulse methylprednisolone may be used to achieve rapid control [35]. The tapering schedule is often individualized, yet a precipitous reduction is associated with a higher risk of relapse [21,42,43].

Despite their efficacy in inducing remission, glucocorticoids are not a long-term solution. Relapse rates after steroid withdrawal range from 30% to 50%, and repeated courses may lead to cumulative toxicity [44]. In fact, a specific tool has been designed to evaluate glucocorticoid-induced toxicity, thereby ensuring that the corticosteroid-sparing strategy positively impacts the patient [45].

Maintenance with low-dose glucocorticoids has been proposed to reduce relapse risk [21]. Consequently, the need for steroid-sparing strategies has become a central goal in IgG4-RD management, motivating the use of conventional immunosuppressants and the exploration of targeted therapies.

### 4.2. Traditional Immunosuppressive Agents

Conventional immunosuppressive agents, including azathioprine, mycophenolate mofetil (MMF), and methotrexate, have been commonly used in IgG4-RD as glucocorticoid-sparing therapies or as maintenance agents following initial remission. Despite the lack of evidence, the latter two have traditionally been the most widely used in the most important patient cohorts [46]. Their use is largely based on extrapolation from other autoimmune conditions, moreover in the biliopancreatic type in IgG4RD.

Mycophenolate mofetil, which selectively inhibits inosine monophosphate dehydrogenase in activated lymphocytes, is another widely used option, particularly in patients with renal, orbital, or pulmonary involvement [47]. One prospective study showed that adding MMF to low-dose corticosteroids reduced the relapse rate after one year [48]. Similarly, two clinical trials have been conducted on cyclophosphamide and leflunomide, both reporting lower recurrence rates when added to glucocorticoid treatment [49,50]. Even though this combined effect has been demonstrated in a network meta-analysis [51], the percentage of recurrences and the potential additional toxicity of immunosuppressants make it necessary to consider alternative options.

Given these limitations, conventional immunosuppressants are often reserved for patients with mild-to-moderate disease activity or as adjuncts when biologic therapies are not available or contraindicated. The lack of robust data supporting their role has further highlighted the need for more effective and targeted treatment options, which are explored in the following sections.

### 4.3. Rituximab

Rituximab, a chimeric monoclonal antibody targeting the CD20 antigen on B cells, has become very beneficial in the management of IgG4-RD, particularly in patients with relapsing, refractory, or multi-organ disease. Its efficacy in depleting circulating CD20^+^ B cells—and indirectly reducing disease-associated CD20^−^CD27^+^CD38^++^ plasmablasts—makes it uniquely suited to interrupt the pathogenic B-cell cascade that characterizes IgG4-RD. It depletes B cells by complement-dependent cytotoxicity and antibody-dependent cell-mediated cytotoxicity.

The first major evidence of rituximab’s efficacy in IgG4-RD came from the prospective, open-label trial by Carruthers et al. [52], which demonstrated clinical improvement in 97% of patients after two infusions of 1000 mg, given 15 days apart. Improvements were observed across multiple organ systems, with reductions in serum IgG4 levels, plasmablast counts, and IgG4-RD Responder Index (RI) scores. Previously, several case series were published that received a very favorable response, given that the biological parameters and clinical features improved [53,54]. Subsequent retrospective series have reinforced these findings, with many patients achieving remission even after failure of conventional immunosuppressants or corticosteroids [12,40,55,56,57,58,59,60,61]. Several meta-analysis reinforce the usefulness of rituximab in the treatment of IgG4, despite the fact that it is currently an off-label drug [43,51,62]. Table 1 shows the main characteristics of the different clinical studies conducted with rituximab.

Rituximab is currently considered a maintenance therapy with a fixed-interval scheme [58]. Despite its high efficacy, several questions remain regarding rituximab’s optimal use. There is no consensus on the optimal maintenance strategy. Some clinicians adopt a fixed re-dosing approach every six months to prevent B-cell reconstitution, which has been associated with longer relapse-free survival [55]. In contrast, others treat only upon clinical or serological relapse [35]. A plethora of protocols exist; nevertheless, the most widely accepted is the 1 g dosage administered in two-week intervals [43]. The absence of validated biomarkers to guide retreatment adds uncertainty. Nonetheless, Lanzillotta et al. [63] demonstrated that incomplete depletion of total CD19^+^, naïve, or memory B cells at six months post-infusion was associated with significantly shorter relapse-free survival (median: 19 vs. 38 months for CD19^+^ cells, *p* = 0.02) and higher relapse rates at both 12 and 24 months. Notably, the complete depletion of B cells six months after rituximab was only achieved in a minority of patients (30% for CD19^+^, 39% for naïve, and 42% for memory B cells). These findings suggest that assessing B cell subpopulations by flow cytometry might offer a practical approach to personalize rituximab re-administration schedules, potentially reducing overtreatment and improving long-term disease control. The same group also identified that the persistence of circulating Tfh cells after rituximab treatment is related with relapse [64]. Despite this, the detection of these cells is more challenging in clinical practice.

Moreover, although generally well tolerated, rituximab carries risks of infusion reactions, hypogammaglobulinemia, and opportunistic infections, particularly in older or comorbid patients [62]. Recently, patients with autoimmune diseases in the context of the COVD-19 pandemic who received rituximab had more severe infections with worse prognoses [65].

Some patients demonstrate partial or non-durable responses, raising the possibility of underlying disease mechanisms not fully addressed by CD20^+^ B-cell depletion alone [40]. This has stimulated interest in therapies targeting other B-cell populations, such as CD19^+^ cells or plasmablasts, as well as those interfering with T-cell and cytokine pathways.

## 5. Emerging Therapies and Novel Targets

Advances in the understanding of the IgG4-RD pathophysiology and the limitations of current therapy have opened up new avenues for targeted treatments. Many of these therapies target mechanisms upstream or downstream of CD20^+^ B-cell depletion, providing a more precise and profound modulation of immune activity. The following subsections explore key emerging therapies under investigation or in the early stages of clinical use (Table 2).

Data on efficacy and safety were extracted from published articles, trial registries (e.g., ClinicalTrials.gov), or manufacturer press releases where appropriate. For trials without peer-reviewed results, only publicly available information is shown. FcRn: neonatal Fc receptor; FDG-PET: [18F]-fluorodeoxyglucose positron emission tomography; IgG4-RD: IgG4-related disease; IL: Interleukin; JAK/STAT pathway: Janus kinase/Signal Transducer and Activator of Transcription; MRI: Magnetic resonance imaging; NA: not applicable.

### 5.1. Inebilizumab

Inebilizumab is a humanized, afucosylated IgG1 monoclonal antibody targeting CD19, a surface antigen expressed on a broader range of B-lineage cells than CD20, including pre-B cells, memory B cells, and plasmablasts. By depleting CD19^+^ cells, inebilizumab may achieve a more profound and sustained suppression of the B-cell compartment, particularly affecting the disease-associated plasmablast population that persists after rituximab therapy [71].

This agent has shown efficacy in trials for other immune-mediated diseases with strong B-cell involvement, such as neuromyelitis optica spectrum disorder (NMOSD), multiple sclerosis and systemic sclerosis [72]. The results of the MITIGATE trial showed that inebilizumab produced greater improvements in reducing disease relapse than the placebo group and decreased the need for chronic glucocorticoid use [36]. This trial randomized 135 patients with active IgG4-RD at a 1:1 ratio to receive either inebilizumab (at a dose of 300 mg) or a placebo intravenously on days 1 and 15, and again at week 26, during the 52-week trial period. Glucocorticoids, which the enrolled subjects were taking at the time of trial entry to treat a recent flare-up of the disease, had to be tapered off by week 8. The cumulative dose of glucocorticoids differed significantly between the two groups: 188.3 mg in the inebilizumab arm and 1384.5 mg in the placebo group. Glucocorticoid withdrawal was more frequent in the inebilizumab group (90%) than in the placebo group (37%).

The potential advantages of inebilizumab include more profound B-cell depletion, longer duration of remission, and lower relapse rates. This treatment’s efficacy and these promising results are largely due to the inhibition of CD19 cells, which extends to B precursors. However, due to its greater potency, there is a higher risk of side effects, as evidenced in the trial (18% vs. 9%), which warrants careful long-term safety monitoring [36]. The broader anti-CD19 effect may offer therapeutic benefits beyond those of rituximab. Furthermore, the absence of direct comparative studies with rituximab and the lack of long-term real-world data limit definitive conclusions regarding its relative benefit and safety profile in clinical practice. Real-world evidence is needed to determine whether inebilizumab offers any therapeutic advantage in the long-term management of IgG4-RD.

### 5.2. Obexelimab

Obexelimab (formerly XmAb5871) is a novel biologic agent designed to modulate B-cell function through a dual mechanism: it binds to CD19 on B cells and simultaneously engages the inhibitory Fc gamma receptor IIb (FcγRIIb). This co-engagement mimics a natural negative feedback loop, resulting in the suppression of B-cell activity without depleting the cells themselves [73]. Unlike rituximab or inebilizumab, obexelimab does not induce B-cell cytotoxicity, but instead maintains immune regulation by preserving B-cell numbers while preventing their pathological activation.

The rationale for obexelimab in IgG4-RD arises from its ability to target pathogenic B-cell subsets, particularly memory B cells and plasmablast precursors, without causing prolonged immunosuppression or hypogammaglobulinemia. In early-phase systemic lupus erythematosus (SLE) trials, obexelimab demonstrated reductions in B-cell activation markers and disease activity, thereby supporting its immunomodulatory potential [74,75].

In IgG4-RD, a phase II open-label study evaluated obexelimab in patients with active disease. Preliminary results suggested clinical benefit, with reductions in the IgG4-RD Responder Index, decreased serum IgG4 levels, and stabilization of affected organ function. Importantly, obexelimab was well tolerated, with a safety profile distinct from that of B-cell depleting agents. In this trial, obexelimab led to a reduction in circulating B cells, and B-cell counts increased after obexelimab withdrawal in most patients [38]. These findings should be interpreted with caution given the small sample size and the absence of long-term follow-up data.

However, long-term efficacy, relapse prevention, and durability of response remain to be fully evaluated in an ongoing phase III trial (NCT05662241) [66]. If this study confirms these benefits, obexelimab could provide a safer alternative to other B-cell-depleted therapies, particularly for patients who are fragile, at risk of infection, hypogammaglobulinaemic, or who have experienced failure of prior B-cell depletion.

### 5.3. Bruton’s Tyrosine Kinase

BTK is a pivotal component of B-cell receptor signaling that plays a key role in the activation, proliferation and survival of B cells. Inhibiting BTK disrupts B-cell receptor signaling and downstream pathways, potentially reducing the generation of autoreactive B cells, antibody production, and pro-inflammatory cytokines. BTK is also expressed in myeloid cells, where it contributes to Fc receptor signaling, suggesting a broader immunomodulatory role. Zanubrutinib is a second-generation BTK inhibitor that has higher specificity for BTK with reduced off-target kinase inhibition which leads to reduced toxicity [76]. It is a frontline treatment in hematologic malignancies like relapsed or refractory chronic lymphocytic leukemia [77]. Zanubrutinib is under consideration for off-label use and merits further investigation in autoimmune diseases, including IgG4-RD (NCT04602598) [67].

Among BTK inhibitors, rilzabrutinib, a reversible covalent oral agent, is currently being evaluated in a phase 2 clinical trial in patients with IgG4-RD (NCT04520451) [78]. In preliminary results from this proof-of-concept study, 70% of patients treated with rilzabrutinib remained flare-free and off corticosteroids or immunosuppressants at week 52. Most adverse events were mild and self-limited [37]. These findings support its potential as an oral, steroid-sparing option in IgG4-RD. The development of BTK inhibitors for IgG4-RD offers a promising therapeutic avenue, particularly for patients with relapsing or steroid-dependent disease, or for those who are intolerant to B-cell-depleting agents.

### 5.4. Other Emerging Therapies

Based on the growing understanding of the immunopathogenesis of IgG4-RD and the diverse clinical phenotypes described above, several molecular and cellular pathways have emerged as potential therapeutic targets. These insights have led to the exploration of novel immunomodulatory agents beyond conventional B cell–directed strategies. Advanced therapies, including biologics and small-molecule inhibitors that target specific cytokines, costimulatory molecules, or intracellular signaling routes, are currently being investigated. Though clinical evidence for many of these approaches remains scarce, their mechanisms of action offer promising steroid-sparing potential and may provide tailored treatment options for patients with refractory, relapsing, or fibrotic forms of the disease.

Efgartigimod, a selective antagonist of the neonatal Fc receptor (FcRn), promotes the degradation of circulating IgG antibodies by blocking their recycling, thus reducing overall IgG levels, including IgG4. This mechanism has shown efficacy in other IgG-mediated autoimmune diseases and may offer a non–B cell-depleting strategy for patients with relapsing or steroid-resistant disease [79]. There is only one case report of the use of efgartigimod in an IgG4-RD patient with spinal meningitis who had a poor response [80]. Currently, a single trial is underway with this molecule (NCT07025330) [68].

Abatacept, a fusion protein composed of the extracellular domain of CTLA-4 linked to an IgG1 Fc fragment, inhibits CD80/CD86–CD28 costimulatory signaling and thereby prevents full T-cell activation. Given the critical role of Tfh and Th2 cells in the immunopathogenesis of IgG4-RD, abatacept may offer therapeutic benefit in cases where T-cell–driven fibrosis plays a central role. Anecdotal reports have described its use in rituximab-refractory cases, such as Mikulicz’s disease with autoimmune pancreatitis [81]. More recently, a proof-of-concept, open-label study evaluated abatacept in 10 patients with active IgG4-RD, showing that 30% achieved complete remission by week 24 [39]. While the drug was generally well tolerated, these findings underscore the modest efficacy and suggest a potential role for abatacept as part of combination strategies or in specific subsets of patients rather than as monotherapy.

Dupilumab is a fully human monoclonal antibody that blocks the shared receptor component for IL-4 and IL-13, two central cytokines in Th2-mediated inflammation. These cytokines play key roles in promoting B-cell class switching to IgG4 and IgE, recruitment of eosinophils, and tissue fibrosis, as previously discussed in the immunopathogenesis section. Originally developed for atopic diseases such as asthma, atopic dermatitis, and chronic rhinosinusitis with nasal polyposis, dupilumab has shown promise in modulating immune responses in diseases with Th2-skewed profiles, an axis that is becoming increasingly implicated in subsets of IgG4-RD. In IgG4-RD, evidence of elevated IgE, peripheral eosinophilia, and tissue infiltration by Th2 cells supports the presence of a type 2 inflammatory environment, at least in some patients [31]. Targeting this pathway may therefore offer a novel, non-B-cell–directed approach to treatment. However, data on the use of dupilumab in IgG4-RD are still very limited. Anecdotal reports and isolated case series suggest a potential benefit, particularly in patients with allergic phenotypes, overlapping eosinophilic diseases, or corticosteroid-dependent disease [69]. The favorable safety profile of dupilumab and its non-immunosuppressive mechanism may be especially attractive for patients at risk for infections or those with contraindications to B-cell–depleting therapies. Nevertheless, its role in IgG4-RD remains investigational. Given the limited data, we believe further studies should clarify which phenotypes may benefit from dupilumab, its effect on fibrosis and plasmablasts, and its role in treatment algorithms.

Tocilizumab, a monoclonal antibody targeting the IL-6 receptor, has shown promise in select cases of IgG4-RD, particularly those with vascular involvement such as aortitis or retroperitoneal fibrosis [82]. Vascular manifestations of IgG4-RD often exhibit a more inflammatory profile, with increased IL-6 expression documented both systemically and within affected tissues [83]. IL-6 production appears to be driven by activated fibroblast-like cells within the vascular wall, suggesting that IL-6 blockade may be particularly beneficial in inflammation-driven phenotypes, although its efficacy in fibrotic, late-stage lesions remains limited. Interestingly, the role of IL-6 in IgG4-RD partially overlaps with that seen in idiopathic multicentric Castleman disease (iMCD), a recognized mimicker of IgG4-RD and an exclusion criterion in the 2019 ACR/EULAR classification criteria [17]. Both entities may share clinical and histopathological features, such as lymphoplasmacytic infiltration and elevated serum IgG4 levels. However, iMCD is typically driven by pathogenic IL-6 overproduction, and the presence of constitutional symptoms, polyclonal hypergammaglobulinemia, anemia, or thrombocytosis should raise suspicion [84]. In this sense, tocilizumab’s efficacy in iMCD supports its rationale in selected IL-6–rich subtypes of IgG4-RD, although current data in IgG4-RD remain limited to case reports and small series [82]. Further studies are warranted to validate biomarkers that may identify patients likely to benefit from IL-6 inhibition in IgG4-RD.

Finally, JAK inhibitors such as tofacitinib and baricitinib have been proposed as oral immunomodulatory agents capable of targeting multiple pro-inflammatory pathways implicated in IgG4-RD. By interfering with the JAK/STAT signaling cascade downstream of several cytokine receptors (e.g., IL-6, IL-4, IFN-γ), JAK inhibition offers a mechanistically broad approach that may benefit patients with multi-organ involvement or a fibrotic phenotype, such as retroperitoneal fibrosis [70]. Two randomized clinical trials are currently evaluating the efficacy and safety of tofacitinib (NCT05625581) and baricitinib (NCT05781516) in IgG4-RD.

## 6. Conclusions

Advances in the immunopathogenesis of IgG4-RD have led to the development of targeted therapies that go beyond conventional immunosuppression. Glucocorticoids remain the cornerstone of induction therapy, providing a rapid response, but they are associated with the potential for relapse and the development of cumulative toxicity. Rituximab has proven effective as a steroid-sparing agent in cases that are refractory or relapse, though concerns remain regarding the optimal maintenance treatment and long-term safety. Notably, inebilizumab is the first biologic agent to demonstrate superiority over a placebo in a phase 3 randomized controlled trial, which could mark a leap forward in treatment. Ongoing research into anti-CD19 agents, BTK inhibitors, JAK inhibitors and other targeted therapies promises to enable more tailored and sustained disease control. The transition from non-specific immunosuppression to targeted interventions, based on the immunobiology of the disease, represents a paradigm shift in IgG4-RD. Furthermore, it establishes this condition as a reference model for developing personalized therapeutic strategies for immune-mediated disorders. The multisystem complexity of IgG4-RD underscores the need for internists with an integrative perspective, capable of coordinating multiple specialties and translating molecular advances into clinical practice. This approach not only optimizes patient outcomes but also redefines the role of the clinician within translational medicine.

## Figures and Tables

**Table 1:** Summary of the main studies on the use of rituximab in IgG4-related disease.

Study/Trial	Study Design	Protocol	Patients (*n*)	Complete Response	Relapses	Follow-Up Time
Khosroshahi et al. (2010) [53]	Retrospective	RTX 1 g 2 weeks apart	4	NA	NA	NA
Khosroshahi et al. (2012) [54]	Retrospective	RTX 1 g 2 weeks apart	10	90%	NA	NA
Carruthers et al. (2015) [52]	Open label trial	RTX 1 g 2 weeks apart	30	47%	10%	12 months
Wallace et al. (2016) [12]	Retrospective cohort	RTX 1 g 2 weeks apart	60	95%	37%	9 months
Ebbo et al. (2017) [55]	Retrospective cohort	Group 1: no RTX Group 2: RTX administered before relapse (variable doses)	33	93.5%	41.9%	25 months
Majumder et al. (2018) [56]	Retrospective cohort	Group 1: RTX induction 2 weeks apart or 375 mg/m^2^/week × 4 weeks Group 2: RTX induction and maintenance every 2–6 months	33	86%	45% group 1 11% group 2	Aprox. 30 months
Urban et al. (2019) [57]	Retrospective cohort	RTX 1000 mg 2 weeks apart or 375 mg/ m^2^/week × 4 weeks	20	75%	15%	38 months
Campochiaro et al. (2020) [58]	Retrospective cohort	RTX 1 g 2 weeks apart in each group (1: induction only, 2a: induction and maintenance 1 g 2 weeks apart every 6 months, 2b: induction and maintenance 1 g every 6 months)	14	NA	71% group 1 vs. 0% group 2	Median 26 months (group 1), 19 months (group 2a), 21 months (group 2b)
Della-Torre et al. (2021) [59]	Prospective	RTX 1 g 2 weeks apart	38	60%	36%	9 months
Backhus et al. (2021) [40]	Retrospective	RTX 1000 mg 2 weeks apart or 375 mg/m^2^/week × 4 weeks	13	NA	61%	71 months
Le Cosquer et al. (2022) [60]	Retrospective	375 mg/m^2^/week × 4 weeks	15			
Colquhoun et al. (2024) [61]	Retrospective cohort	RTX 1 g 2 weeks apart Re-treatment 2 × 1 g or 1 × 1 g	45	22.5%	18%	30 months

NA: not applicable; RTX: rituximab.

**Table 2:** Emerging treatments in IgG4-related disease.

Treatment	Mechanism of Action	Trial/Study	Clinical Rationale	Primary Outcome	Efficacy/Status
Inebilizumab [36]	Inhibition CD-19+ B cells	NCT04540497 MITIGATE Phase 3 Placebo-con-trolled RCT	Broader B cell depletion than anti-CD20; targets plasmablasts and plasma cells involved in IgG4 production.	Time to relapse	Relapse rate: 10% vs. 60% HR: 0.13 (95% CI, 0.06–0.28; *p* < 0.001) Serious AEs: 18% vs. 9%
Obexelimab [66]	Inhibition CD19 and FcγRIIb co-ligation	NCT05662241 INDIGO Phase 3 Placebo-con-trolled RCT	Inhibits B cell activation without depletion; potential to suppress pathogenic IgG4-producing B cells	Time to relapse	Active, no longer enrolling
Zanubrutinib [67]	Selective Bruton’s tyrosine kinase inhibitor	NCT04602598 Phase 2, Single-Site, Open-Label	Inhibiting B cell receptor signaling, reducing activation and differentiation of B cells and plasmablasts; potentially decreases autoantibody production and inflammatory responses in IgG4-RD.	Volume of the submandibular/lacrimal glands on PET-MRI	Trial completed; results pending publication
Rilzabrutinib [37]	Reversible covalent inhibitor of Bruton’s tyrosine kinase	NCT04520451 Phase 2a, multicenter, open-label Ongoing Phase 3	Inhibiting B-cell activation, possibly decreasing autoantibody production, preventing FcγR-mediated phagocytosis in the spleen and liver, and reducing chronic inflammation	Time to relapse	70% flare-free at week 52; mild AEs
Efgartigimod [68]	Reduction pathogenic IgG autoantibody levels	NCT07025330 Phase 2a, Single-Site, Open-Label	Blocking FcRn accelerates the catabolism of IgG4, which could potentially reduce the levels of pathogenic immune complexes and serum IgG4 without inducing immunosuppression.	Change in volume on FDG-PET/ MRI organ involvement	No data available yet
Abatacept [39]	CD80/CD86–CTLA4-Ig fusion protein	Single-arm open-label trial	Blocks T cell co-stimulation and B-T interaction; shown benefit in observational cohorts.	Complete remission	Complete remission at 12 weeks in 30% 60% partial remission at 12 weeks, 50% at week 24 80%
Dupilumab [69]	IL-4 and IL-13 receptor alpha subunit	Case reports/early-stage evaluation.	Blocks type 2 cytokines involved in IgG4 switch; promising in Th2-driven IgG4-RD phenotypes	NA	Anecdotal benefit in allergic/Th2 phenotypes
JAK inhibitors [70]	JAK/STAT pathway	Theoretical/experimental	Broad anti-inflammatory effects; may inhibit cytokine cascades contributing to fibrosis and inflammation.	NA	No trials in IgG4-RD to date

NA: not applicable.

## Data Availability

Not applicable.

## Abbreviations

The following abbreviations are used in this manuscript:

ACR	American College of Rheumatology
AE	Adverse event
BAFF	B-cell activating factor
BTK	Bruton’s tyrosine kinase
CD	Cluster of differentiation
EULAR	European League Against Rheumatism
FcRn	Neonatal Fc receptor
FDG	Fluorodeoxyglucose
GC	Glucocorticoid
IFN	Interferon
IgE	Inmunoglobulin E
IgG4-RD	Immunoglobulin G4–related disease
IL	Interleukin
JAK	Janus kinase
PET	Positron emission tomography
RCT	Randomized controlled trial
STAT	Signal transducer and activator of transcription
TFH	T follicular helper
TGF-β	Transforming growth factor-β
TH2	T helper 2
